# Effect of biogenic selenium nanoparticles on ERG11 and CDR1 gene expression in both fluconazole-resistant and -susceptible *Candida albicans* isolates

**DOI:** 10.29252/cmm.3.3.16

**Published:** 2017-09

**Authors:** Nasrin Parsameher, Sassan Rezaei, Sadegh Khodavasiy, Samira Salari, Sanaz Hadizade, Mohammad Kord, Seyed Amin Ayatollahi Mousavi

**Affiliations:** 1 Department of Medical Parasitology and Mycology, School of Medicine, Kerman University of Medical Sciences, Kerman, Iran; 2 Department of Medical Parasitology and Mycology, School of Public Health, Tehran University of Medical Sciences, Tehran, Iran

**Keywords:** *Candida albicans*, *CDR1*, *ERG 11*, Nanoparticles

## Abstract

**Background and Purpose:**

*Candida albicans* is the most common *Candida* species (sp.) isolated from fungal infections. Azole resistance in *Candida* species has been considerably increased in the last decades. Given the toxicity of the antimicrobial drugs, resistance to antifungal agents, and drug interactions, the identification of new antifungal agents seems essential. In this study, we assessed the antifungal effects of biogenic selenium nanoparticles on *C. albicans* and determined the expression of *ERG11* and *CDR1* genes.

**Materials and Methods:**

Selenium nanoparticles were synthesized with *Bacillus* sp. MSH-1. The ultrastructure of selenium nanoparticles was evaluated with a transmission electron microscope. The antifungal susceptibility test was performed according to the modified Clinical and Laboratory Standards Institute M27-A3 standard protocol. The expression levels of the *CDR1* and *ERG11* genes were analyzed using the quantitative real-time polymerase chain reaction (PCR) assay.

**Results:**

The azole-resistant *C. albicans* and wild type *C. albicans* strains were inhibited by 100 and 70 µg/mL of selenium nanoparticle concentrations, respectively. The expression of *CDR1 *and *ERG11* genes was significantly down-regulated in these selenium nanoparticle concentrations.

**Conclusion:**

As the findings indicated, selenium nanoparticles had an appropriate antifungal activity against fluconazole-resistant and -susceptible *C. albicans* strains. Accordingly, these nanoparticles reduced the expression of *CDR1* and *ERG11* genes associated with azole resistance. Further studies are needed to investigate the synergistic effects of selenium nanoparticles using other antifungal drugs.

## Introduction

Over the past decade, *Candida* infections has dramatically increased among the high-risk patients [1]. The excessive use of antifungal agents due to the limited availability of antifungal medications leads to the emergence of drug resistance in pathogenic species [2]. Regardless of drug resistance, the long-term use of antifungal medications has irreparable side effects in patients, which can lead to death in some cases [3, 4]. 

The rate of azole resistance is on a growing trend in *Candida* species. Accordingly, there are extensive biochemical studies highlighting a significant diversity in the mechanisms conferring resistance to azoles. Some of these mechanisms include changes in the efflux system and mutation in ergosterol biosynthesis pathway genes [5]. The increased mRNA levels of *Candida *drug resistance gene family (*CDR*) and multi-drug resistance (MDR) have been associated with azole resistance [5, 6]. In addition, the overexpression of *CDR1 *and *CDR2 *in *C. albicans *is associated with cross-resistance to the azoles [7]. 

Ergosterol biosynthesis pathway is another pathway of azole resistance. The overexpression of *ERG11* gene leads to an increase in the target drug. Therefore, a higher concentration of drug is required to react to all of enzyme molecules present in the cells, and thereby decrease the susceptibility to azole in *Candida* species [8]. The advent of this drug resistance necessitates the development of new antifungal agents to overcome the resistant strains. 

Nanoparticles (NPs) are a new generation of antimicrobial agents that have been highly studied in the last two decades [9-11]. Among them, metal NPs and metal oxide have unique properties and are considered as antimicrobial agents [12]. Selenium is one of the essential elements for human health that improves the immune system and exerts anti-cancer effects. Nevertheless, there are problems with the pharmaceutical application of selenium for the treatment of infections. The nanotechnology recommends the use of this element for nutritional and pharmaceutical purposes [13]. 

On the other hand, selenium nanoparticles (Se NPs), as strong antioxidants, are much less toxic than selenium. Moreover, they have high power in scavenging free radicals; consequently, they can be used as a natural antioxidant [14]. Selenium sulfide has long been known as an antifungal agent used as anti-dandruff [15]. However, few studies have been published on the antifungal effects of Se NPs. 

The expression of *ERG11* and *CDR* genes have been identified as the mechanisms responsible for resistance to azole in *Candida* species. Regarding this and given the alterations in the structure of these two genes, the present study aimed to investigate the inhibitory effect of biogenic Se NPs on fluconazole-resistant *C. albicans*. In addition, the alternation in *ERG11* and *CDR1* gene expression was assessed in fluconazole-resistant *C. albicans* using the real-time polymerase chain (PCR) reaction technique.

## Materials and Methods

***Candida albicans*** ***strains***

Fluconazole-resistant *C. albicans* (ATCC 76615) and fluconazole-susceptible *C. albicans* (ATCC 10231) were used to test antifungal susceptibility and gene expression by real-time PCR.

***Preparation of stock nano-silver solution***

The biosynthesis of Se NPs was accomplished using Bacillus sp. MSh-1 strain, which was previously isolated from the Caspian Sea (located in the northern part of Iran) and identified by 16S rDNA gene analysis technique (GenBank accession number: GU183144.1), based on a method described by Shakibaie et al. [16]. Briefly, selenium dioxide powder (Merk, Germany) was dissolved in sterilized distilled water, and then filtered by a 0.22 μm diameter pore. In the next step, 1 mL of this solution was added to 99 mL nutrient medium; subsequently, 1 mL of *Bacillus *sp*.* Msh-1 suspension was added. 

After 14 h, the obtained solution was centrifuged for 10 min at 5000 rpm, and the supernatant was removed. Sediment was washed twice with 0.9% sodium chloride. The cells were then broken down by the liquid nitrogen and sonication (5 min/100 W). Subsequently, 4 ml of the above suspension was added to 2 ml ethanol and mixed vigorously. The tubes were centrifuged at 3000 rpm for 5 min and cooled for 24 h at 4°C to separate the two phases. After the gentle removal of the supernatant, the residual nanoparticles were washed with the chloroform, ethanol 70%, and distilled water, respectively. 

***Nanoparticle structure***

The size and morphology of Se NPs were examined by using a transmission electron microscope (TEM, Zeiss Supra 55 VP TEM, operated at 100 KV). The chemical properties of nanoparticles were also characterized by the energy-dispersive X-ray spectroscopy technique [17].

***Antifungal susceptibility test***

Fungistatic activity and minimum inhibitory concentrations (MICs) of Se NPs against *C. albicans* strains were determined according to the recommend-dations stated in the Clinical and Laboratory Standards Institute M27-A3 and M27-S4 documents [18]. An RPMI-1640 medium buffered at pH 7.0 with 3-N-morpholino propanesulfonic acid was used as the culture medium. The inoculum size of *C. albicans* was 0.5-2.5×10^3^ cells/mL. 

The concentration of Se NPs in the dispersion ranged within 10-200 µg/mL. Subsequently, 100 μL of yeast suspension and 100 μL of each concentration of Se NPs were added to the respective well of microtiter plates. The microdilution plates inoculated at 35^o^C for 48 h. The lowest concentration of the Se NPs, inhibiting the visible growth of microorganisms, was considered as the MIC. *C. parapsilosis* ATCC 22019 standard strain was used as quality control.

***RNA extraction and complementary*** ***DNA synthesis ***

We extracted RNA from the last dilution of the drug based on MIC, and also from the positive control sample. Afterwards, 6×10^8^ cells of the fungal suspension were centrifuged for 2 min/12000 g, and the supernatant was removed. RNA was extracted using the RNA extraction kit (Gene, JET RNA purification kit, fermentase, Germany). After centrifugation, the sediment was stored at -20^o^C until use. 

The complementary DNA (cDNA) was synthesized according to the manufacturer's recommendations using a cDNA synthesis kit (Fermentas, USA). The materials used for making cDNA included 0.9-1.5 μL RNA template, 1 μL of random hexamer primer, 9.5-10.1 μL DW, 4 μL 5x reaction buffer, 1 μL RiboLock RNase Inhibitor, 2 μL of 10 mM dNTP Mix, and 1 μL reverse transcriptase in a final volume of 20 μL. 

***Real-time polymerase chain reaction***

Real-time PCR was performed using a Corbett Rotor-Gene 3000 (Corbett Robotics, Australia), and SYBR Premix Ex Taq II (BioFact, Daejeon, Korea). The sequence of primers and their characteristics are illustrated in Table 1. All PCR mixtures contained 10 μL SYBR Premix Ex Taq II (2×), 2 μL of first strand cDNA, 1 μL of each specific primers (4 pmol) and 6 μL of diethyl pyrocarbonate water in a final volume of 20 μL. The amplification was initiated at 95°C for 15 min, followed by 45 cycles of denaturation at 95°C for 30 sec, annealing at 60°C for 40 sec, and extension at 72°C for 30 sec. 

**Table 1:** Nucleotide sequences of *CDR1 *and *ERG11 *primers

**Name**	**nt**	**GC%**	**Tm**	**Sequence**	**GeneBank**
*CDR1-S*	*20*	*50*	*53.65*	*5’GGTGCTAATATCCAATGTTGG’3*	*X77589.1*
*CDR1-AS*	*20*	*50*	*50.22*	*5’GTAATGGTTCTCTTTCAGCTG’3*	
*ERG11-S*	*20*	*45*	*59.88*	*5’CAGAAAAGTGGCGTTGTTGA’3*	*KM875729.1*
*ERG11-AS*	*20*	*45*	*59.69*	*5’GCAGCATCACGTTTCCAATA’3*	

The expression of all genes was normalized to the housekeeping gene *β actin.* Standard curves for each gene were established with five serially diluted cDNA, obtained from the cells grown to mid-logarithmic phase, using specific primers under the appropriate PCR conditions. Experiments under each condition were performed in duplicate, and negative control (water as template) was included in each run. The *CDR1 *and *ERG11 *gene expression was analyzed with REST software (2009; Version 2.0.7). The software uses the comparative Ct method (ΔΔCt) to analyze data. 

## Results

**Figure 1:**
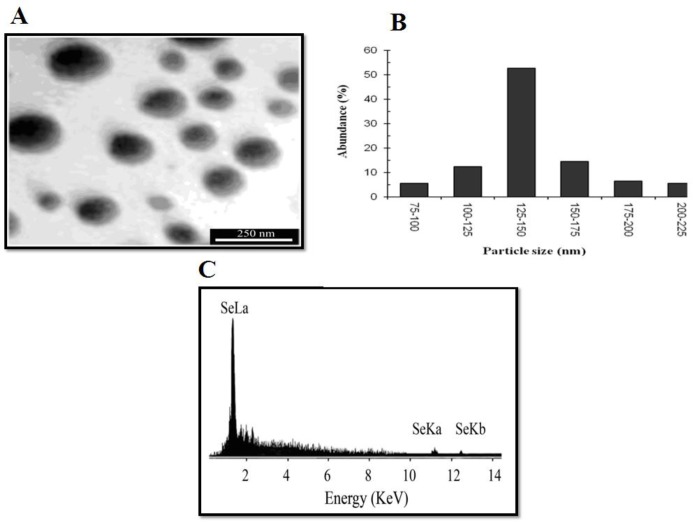
Synthesis and characterization of selenium colloidal nanoparticles synthesized by using *Bacillus* sp. MSh-1, A) Transmission electron micrograph of pure selenium nanoparticles, B) Particle size distribution histogram of the biogenic selenium nanoparticles, C) Energy-dispersive X-ray images of purified selenium nanoparticles

Spherical Se NPs were produced in a range of 80-200 nm by *Bacillus sp*. Msh-1 based on the transmission electron micrograph from pure Se NPs, and nanoparticles in the range of 125-150 nm were the most frequent ones. The energy-dispersive X-ray spectroscopy micro analysis of pure Se NPs showed three absorption peaks consisting of SeLα (1.37 keV), SeKα (11.22keV) and SeKβ (12.49 keV) (Figure 1). The MIC for fluconazole-resistant *C. albicans* and fluconazole-susceptible *C. albicans *in antifungal susceptibility test for Se NPs were 100 and 70 µg/mL, respectively. 

Fluconazole-resistant and -susceptible *C. albicans* strains were shown to reduce the expression of *CDR1* and *ERG11* genes after exposure to 70 and 100 µg/mL Se NPs, respectively. The results of *CDR1* and *ERG11 *gene expression are displayed in Table 2. In fluconazole susceptible-*C. albicans*, *CDR1* and *ERG11* were down-regulated in the sample group by the mean factors of 0.067 and 0.038 expression rates, respectively. Similarly, in fluconazole-resistant *C. albicans, CDR1* and *ERG11 *were down-regulated in the sample group by the mean factors of 0.022 and 0.027 expression rates, respectively.

**Table 2:** Results of *CDR1* and *ERG11* genes expression in fluconazole-resistant and -susceptible *C. albicans*

**Candida species**	**Gene**	**Type**	**Reaction efficiency**	**Expression**	**Result**
Fluconazole-resistant *C. albicans*	*CDR1*	TRG	0.69	0.022	Down
	*ERG11*	TRG	1.0	0.027	Down
Fluconazole-susceptible *C. albicans*	*CDR1*	TRG	0.64	0.067	Down
	*ERG11*	TRG	1.0	0.038	Down

TRG: target, REF: reference, Down: down-regulation

## Discussion

The use of nanotechnology has increased in many medical fields, especially drug delivery [12]. The synthesis of nanoparticles by microorganisms and plant extracts is suggested as a suitable method, compared to the physical and chemical methods [13]. Unique properties, such as high surface-to-volume ratio and their nanoscale size, are the advantages of nanoparticles. This high surface-to-volume ratio of the nanoparticles provides more active sites for interacting with biological entities, such as cells [19]. 

To date, the antifungal effects of different nanoparticles, such as iron NPs, silver NPs, and gold NPs, have been studied [20-23]. The Se NPs increase the efficiency of glutathione peroxidase and thioredosin reductase [24] . Nano selenium is also reported as an antioxidant with reduced risk of direct toxicity on cells [25]. The antibacterial, antiviral, and antioxidant activities of Se NPs, in addition to their lower toxicity, have introduced them as interesting compounds in nano-biotechnology [26, 27]. 

In this study, the antifungal activity of Se NPs against *C. albicans* as a model for fungi was investigated. The results of the study showed that the Se NPs produced by *Bacillus MSH-1* had antifungal activity against *C. albicans*. Limited studies have been carried out on the Se NPs. The anti-biofilm activity of biogenic Se NPs and selenium dioxide against the clinical isolates of *Staphylococcus aureus*, *Pseudomonas aeruginosa*, and *Proteus mirabilis* has been demonstrated [28]. 

Kazempour *et al.* investigated the antifungal activity of Se NPs, produced by Klebsiella pneumonia, against *Aspergillus niger* and *C .albicans*. They reported the Se NPs MIC values of 250 and 2000 µg/mL for A. niger and C. albicans, respectively [29]. In addition, Shakibaei *et al*. showed the antifungal effects of Se NPs synthesized by *Bacillus* species on *Aspergillus fumigatus* and *C. albicans*. In the mentioned study, the MICs of Se NPs against *C. albicans* and *A. fumigatus* were 70 and 100 μg/mL, respectively [30], which is consistent with our results.

Several genes associated with azole resistance (e.g., *CDR1*, *CDR2*, *MDR1*, *ERG3*, *ERG6*, *ERG11*, *ERG9*, *RTA2,* and *NAG2*) have been identified in *Candida *species. Each of these genes promotes the resistance of the organism to antifungal drugs with different molecular mechanisms [31-33]. All of these results indicate that Se NPs can be effective in the fungus with different mechanisms, compared to the conventional antifungal agents. 

According to the results of this study, Se NPs could reduce the resistance of *C. albicans* to antifungal drug by decreasing the expression of the drug-related genes. In our study, the real-time PCR revealed the down-regulation of *CDR1* and *ERG11* genes for *C. albicans* exposed to Se NPs with concentrations of 70 and 100 μg/mL, respectively. 

## Conclusion

In conclusion, Se NPs reduced the expression of *CDR1* and *ERG11* genes. The down-regulation of these genes can decrease the resistance of *Candida* strains to azole. Common antifungal agents that are currently prescribed for the treatment of various fungal infections, have limitations, such as toxicity and drug resistance. According to the obtained results, the synthetized Se NPs can be an appropriate agent against *C. albicans*; moreover, they can facilitate overcoming the azole resistance. However, it is required to conduct more in vitro and in vivo investigations in this regard.
